# Reproductive Management of Rams and Ram Lambs during the Pre-Breeding Season in US Sheep Farms

**DOI:** 10.3390/ani11092503

**Published:** 2021-08-26

**Authors:** Martin G. Maquivar, Sarah M. Smith, Jan R. Busboom

**Affiliations:** 1 Department of Animal Sciences, College of Agriculture, Human and Natural Resources Sciences, Washington State University, Pullman, WA 99164-1067, USA; busboom@wsu.edu; 2 Grant-Adams County, Extension, Washington State University, Moses Lake, WA 98837-9753, USA; smithsm@wsu.edu

**Keywords:** pre-breeding management, breeding soundness evaluation, reproductive health, reproduction, sheep

## Abstract

**Simple Summary:**

Pre-breeding management of rams and their ram lambs is critical for viable, sustainable and profitable farms. A further understanding of the management and selection criteria of the males during pre-breeding enables producers to efficiently use animal resources to maximize the reproductive potential of the animals. The reproductive management of mature rams and their lambs is complex, varying across breeds, farms, and regions of the world. It involves different aspects of animal husbandry, such as genetics, health, nutrition, behavior, economy and physiological and anatomical changes during the non-breeding season. Sound flock management must include an integrative and complete management plan for males and females that provides adequate conditions for the sheep to express their genetic potential and production level. This review article examines some of the key aspects to consider when managing mature rams and ram lambs during the pre-breeding season to ensure proper condition of the males for optimal function during the breeding season.

**Abstract:**

In sheep farms, achieving economic and productive efficiency and sustainability goals is directly related with the reproductive management of the animals. Genetically, the male contribution to the offspring is 50%, but in practical terms, there is a greater potential impact of the ram on reproductive results, because one male has the potential to affect a large group of females and therefore greatly affects the entire flock. Unfortunately, the selection of males for breeding in the US sheep industry has been traditionally conducted based on phenotypical traits, without a genetic/reproductive evaluation, and/or health assessment or records. Therefore, it is important to establish integrative management practices to select the rams and ram lambs to be used in the breeding season. Among these practices are genomic testing, a comprehensive breeding soundness evaluation and assessment of health status and records of the males before the breeding season, to detect and correct potential issues.

## 1. Introduction

To achieve adequate reproductive performance during the breeding season (>95% of the ewes pregnant by the end of the season), it is necessary to implement husbandry practices during the non-breeding season to ensure the proper conditioning of the males and females. The standard ovine breeding season in the northern hemisphere is from September to December, with a successive lambing period occurring from February to May the following year. This rather short timeframe puts a great time constraint on producers in terms of organizing the breeding season as well as maintaining the profitability of their operation.

The reproductive performance of the animals is one of the most important traits, due to its impact on the overall profitability of the flock. Fertility is a particularly crucial trait; it affects the production and economic efficiency of ovine industries. Previous reports suggest that only 70–75% of the males in a flock are reproductively optimal at the beginning of the breeding season [1,2,3]. Therefore, the management of males that are going to be used during the breeding season presents several challenges, not only in terms of the selection and acquisition of animals, but for the general management of males in the pre-breeding, mating and post-mating seasons [4]. Management of the males is necessary to ensure the success of the flock productively and economically and to maximize the longevity of the animals [5]. The reproductive performance of rams and lambs is affected by numerous factors, including the overall health of the animals, nutrition, and the effect of the environment. The present review will focus on the key aspects of the management of rams and lambs before the breeding season in ovine production systems in the United States.

## 2. Reproductive Seasonality and Puberty

In species such as small ruminants raised in the northern hemisphere, environmental factors play a critical role in the regulation of reproductive processes. Among these factors are the amount of light, or the photoperiod. In ovine species, the amount of light plays an important role in regulating the timing of various reproductive processes. A decrease in the amount of natural light results in an increase in the concentration of melatonin during fall and winter months; this has a positive effect on the regulation of the reproductive axis, resulting in an increase in responsiveness of the pineal gland and secretion of melatonin. This affects the neurons located in the hypothalamus and gonadotrophs in the pituitary, leading to endocrinological changes in the gonads that stimulate an increase in the synthesis of steroids such as testosterone. This induces production of viable spermatozoa and results in an increase in sexual behaviors (libido, aggressiveness, etc.) [6,7]. By the beginning of the breeding season (late August to September in the northern hemisphere), resumption of testicular activity and increased concentration of reproductive hormones (GnRH, LH, FSH and Testosterone) are expected [8,9,10].

The age of onset of puberty in ram lambs varies across breeds and climatic conditions [11]. Nevertheless, it is characterized by a series of endocrine events that activate the reproductive axis, which allow the Sertoli and Leydig cells to become responsive to the gonadotropin action (LH and FSH), resulting in the initiation of spermatogenesis. The age at puberty in ram lambs is influenced by the photoperiod, but a more important factor is the plane of nutrition. In fact, early studies by Martin et al. [10] observed that feeding a high protein diet induced an increase in the testicular tissue, consequently advancing the age at puberty. Additionally, puberty can be characterized by anatomical changes at the testicular level, specifically by the increase in testicular tissue. Early studies performed by Yarney et al. [12,13] showed that testicular size and spermatogenic function are positively correlated and that ram lambs with greater testicular size at six months of age had a greater daily sperm output and were mating with ewes more frequently. Depending on the breed of the animal, the onset of puberty, characterized as the presence of motile spermatozoa, can occur as early as 16–18 weeks of age in some breeds, such as Suffolk [14,15]. However, normal ranges for the onset of puberty are between 20 and 28 weeks of age and occur at attainment of 65% of mature body weight [16].

## 3. Selection of Rams and Ram Lambs Prior to the Breeding Season

Unfortunately, the selection of males for breeding in the US sheep industry has been traditionally conducted based on phenotypical characteristics, without a current genetic/reproductive evaluation and/or health records. Currently, the National Sheep Improvement Program [17], established in 1987, records and collects data from 23 breeds of sheep. The main goal is to provide genetic information and evaluations, resulting in estimated breeding values of sires in order to drive better management decisions and to increase the productivity of the flocks.

**Figure 1:**
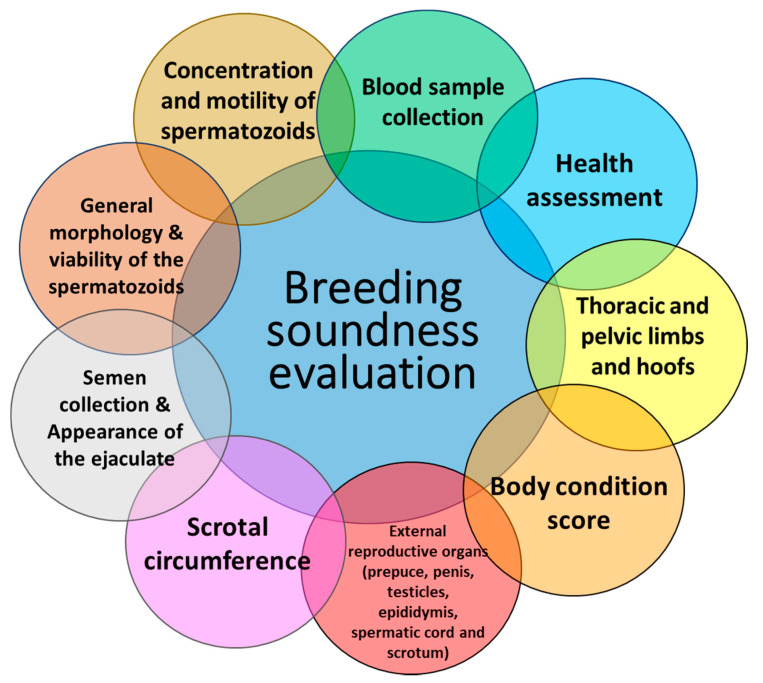
Breeding soundness evaluation of rams and ram lambs prior to the breeding season.

In addition to genetic evaluation and testing, an important tool to select potential breeding rams and ram lambs is through breeding soundness examinations (BSEs). A basic BSE consists of (1) a general clinical evaluation, including inspection of the mouth and teeth and determination of age of the animal (if records do not exist); (2) assessment of the general conformation of the thoracic and pelvic extremities; however special attention is given to the pelvic limbs and hoofs as well as mobility or locomotion scores; (3) evaluation and assessment of the body condition score [18]; (4) inspection of the external reproductive organs (prepuce, penis, testicles, epididymis and scrotum) by manual palpation and, in certain instances, by ultrasonographic evaluation of the testicular and epididymis tissue; (5) measurement of the scrotal size; (6) semen collection and evaluation, including the appearance of the ejaculate (volume, color and presence of abnormal secretions such as blood or pus), general morphology and viability of the spermatozoa (using histological dyes) and concentration and motility of the spermatozoa; (7) in some cases, collection of blood and fecal samples to diagnose/detect important diseases such as parasites and *Brucella ovis*. These aspects of the BSE are usually performed 2–4 weeks prior to the breeding season to determine the ability of the male to reproductively perform; however, in some cases, males have unsatisfactory values for one or more of the aspects of the BSE, and in this situation, a second BSE is recommended at least 2–3 weeks after the first. Figure 1 summarizes the various aspects of the BSE evaluation performed on mature rams (>2 years old) and ram lambs (≤1 year old).

## 4. Breeding Soundness Evaluation

Assessment of the reproductive soundness of the male prior to the mating season could improve the selection criteria for males used for reproductive purposes. Unfortunately, a BSE of rams and lambs is often overlooked because it requires labor, time, trained personnel, and economic inputs from the producers [5], in spite of the fact that the use of males that are reproductively capable will positively impact the overall number of lambs born, lambing rates (defined as the number of lambs/year/ewe), the productive efficiency of the herd, economic resources and the economic return to the producers. A study conducted by Van Metre et al. [3] found that out of 11,804 rams submitted to a BSE, 29% of were considered not optimal for reproduction based on the semen characteristics. The primary reasons for failure were substandard semen parameters, such as low motility (<30%), low concentration of spermatozoa (<2 × 10^9^/mL), an increase in abnormal morphology (>50% abnormalities) of the spermatozoa, and inflammatory causes accompanied by physical abnormalities of the reproductive organs. Among the main causes for culling males from the flock are low reproductive performance in the previous breeding season, overall body condition score and the health status of the animal [19]. Scientific evidence has demonstrated a clear association between the metabolic status of an animal expressed in body reserves/body condition score and reproductive efficiency. In sheep, the photoperiod and the fat reserves play a critical role in the activation of the reproductive organs (Hypothalamus—GnRH—and the hypophysis—FSH and LH—secretion), which impact the ability of the animal to produce spermatozoa [20,21]. Low body condition scores at the time of the BSE have been associated with low reproductive efficiency, and they can increase the risk of failure of the BSE [3]. In general terms, it is recommended to have a BCS of 3.0–3.5 on a 1 to 5 scale (where 1 = an emaciated animal and 5 = an obese animal) at the beginning of the breeding season [22]. Rams often fall below a 3.5 after the breeding season has ended, so it is crucial to have a score of 3.5 approximately 4 weeks prior to the start of the next breeding season as nutrition has a significant impact on reproductive abilities such as semen production [16,17,18,19,20,21]. If males are low in BCS (<3.0) after the breeding season, it is recommended to provide a high-energy ration to increase the body weight, testicular size and number of cells in the germinal layers of the testicle, resulting in increased sperm production during the non-breeding season [19]. Table 1 presents data on the different breeds of sheep around the world; data presented is for pubertal rams. The variation observed among the different breeds of rams can be attributed to the management of the animals, the region in which they are raised and overall nutrition.

**Table 1:** Scrotal size and semen characteristics of different breeds of sheep across the world. Data presented in the table show values for pubertal rams.

Breed	Season of Collection/Age of the Male	Scrotal Circumference (cm)	Spermatozoa Concentration (10^9^/mL)	Semen Volume (mL)	Location of the Study	Reference
Pelibuey	Short days Long days	30.9 ± 0.12 29.59 ± 0.32	4.40 ± 0.16 4.02 ± 0.19	0.86 ± 0.02 0.73 ± 0.05	Mexico	Aguirre et al. [23]
Ile-De-France	Spring Age (years): 1–2 2–3 3–4 >4	Average (range) 28.8 (25–34) 28.5 (26–31) 30.7 (28–33) 35.4 (28–41)	2.5 ± 1.2	1.15 ± 0.6	Morocco	Toe et al. [24]
Fleischschaf Merino Charmoise	September–January	33.74 ± 0.25 35.01 ± 0.23 30.88 ± 2.26	4.12 ± 1.29 4.23 ± 1.47 4.99 ± 1.11	0.78 ± 0.03 0.77± 0.03 0.95 ± 0.22	Spain	Arrebola-Molina et al. [25]
Suffolk	October–November (6–7 month of age)	31.1 ± 0.6	3.96 ± 0.8	Not assessed	Canada	Yarney et al. [12]
Suffolk	Autumn	31.54 ± 0.68	2.78 ± 0.36	1.06 ± 0.05	Brazil	Milczewski et al. [26]
Dorper	Summer (end) Extensive management Intensive management	31.8 ± 0.6 34.1 ± 0.4	1.17 ± 1.23 0.73 ± 1.34	1.1 ± 0.1 1.1 ± 0.1	South Africa	Fourie et al. [27]
Finn Dorset	November–December	29.2 ± 0.6 27.8 ± 0.7	1.3 ± 0.1 1.1 ± 0.2	Not assessed	USA	El-Alamy et al. [28]
Karakul	Autumn	33.3 ± 1.4	4.57 ± 1.66	1.3 ± 0.3	Iran	Kafia et al. [29]
Awassi	19 months of age	Not assessed	4.0 ± 1.6	1.15 ± 0.5	Syria	Salhab et al. [30]
Creole Romney Marsh Hampshire	Rainfall season (September–November)	Not assessed	2.79 ± 2.80 2.16 ± 1.68 2.38 ± 1.63	1.85 ± 0.09 2.29 ± 0.19 2.81 ± 0.17	Colombia	Carvajal-Serna et al. [31]
Targhee	August	33.79 ± 0.62	1.14	Not assessed	USA	Page et al. [32]
Corriedale	Autumn	32.8 ± 0.3	3.5 ± 0.1	0.8 ± 0.1	Argentina	Aller et al. [33]
Katahdin	Dry and Rainy season	35.03 ± 0.26	2.57 ± 1.05	0.6 ± 0.04	Mexico	Cárdenas-Gallegos et al. [34]
Debouillet	Autumn	Not assessed	2.47 ± 2.27	1.8 ± 0.3	USA	O’Neill and Hallford [35]

Another important aspect is the assessment of the scrotal circumference, as it can vary by season and is affected by the body condition score of the animal. It has also been found that sperm production directly and positively correlates to testicular circumference [36]. According to the recommendation by the American Sheep Industry Association [37], ram lambs and adult rams with a scrotal circumference less than 30 cm and 32 cm, respectively, have lower breeding capabilities, and it is recommended that they not be used for breeding purposes. In contrast, ram lambs and adult rams with a scrotal circumference greater than 33 cm and 35 cm, respectively, exhibit greater breeding capabilities. The general criteria proposed by Van Metre et al. [3] consider a ram to be satisfactory as a potential breeder when males between 6 and 13 months of age have a scrotal circumference of 30–35 cm in diameter and males older than 14 months have a circumference of 33–39 cm. There is also evidence suggesting that rams with larger testicles have the potential to sire more prolific ewes [38].

In addition, it is important to detect physical defects in the reproductive tract that can potentially affect the fertility of the male. Among these defects, one of the most common is unilateral or bilateral cryptorchidism. Cryptorchidism is characterized by the failure of one (unilateral) or both testes (bilateral) to descend into the scrotum. If both testicles are affected, the ram is infertile, whereas if one testicle is affected, the ram may be fertile, but the trait can be passed to the offspring, and if detected it should be an automatic reason for culling of the ram [38,39].

Finally, the overall health of the rams and ram lambs should be evaluated prior to the breeding season, with special attention paid to lameness and hoof integrity. Rams and ram lambs should have a locomotion score of 0, where 0 is an animal that has normal locomotion and is not lame, 1 is an animal that has a clear shortening of gait with a clear head movement when the affected limb touches the ground, 2 is an animal that has clear gait shortening with strong head movement and not bearing or supporting any wight on the affected limb, and 3 is an animal that is reluctant to stand or move [40,41]. Another important aspect of the pre-breeding health assessment includes evaluation of the presence of abnormal ocular and nasal discharges, skin wounds, lesions and abscesses in the skin or lymph nodes that could indicate caseous lymphadenitis (*Corynebacterium pseudotuberculosis*) [42], abnormal urinary secretions [43], respiratory problems such as pneumonias and pulmonary abscess [44], scours and fecal soiling that could indicate parasitic or bacterial diseases, ruminal movements as well as the general appearance and behavior of the animal.

## 5. Semen Collection and Seasonality

In mature rams and ram lambs, it has been reported that semen collection performed during the spring and summer results in lower sperm production, increased abnormal spermatozoa, and overall decreased fertility [45]. In a study conducted by Moghaddam et al. [46], seasonal variations in semen quantity and quality were reported. Ten rams were collected during the spring and summer months and had their semen evaluated for volume, total spermatozoa per ejaculate (TSE), semen color, spermatozoa progressive motility, and percentage of live and abnormal spermatozoa. It was found that semen quality and quantity was significantly lower in comparison to the fall and winter months [46]. Similarly, Gomez [47] examined the morphological and physiological reproductive changes produced by rams during the summer months in Washington State (June–August) in comparison to the fall months (September–December) to determine the structural changes in rams during the transition period and whether they were still fertile outside of the standard breeding season. The study included 11 Suffolk-Hampshire crossbred sheep, ranging from one to three years of age. Based on the results of this study, ejaculate from the summer months was considered viable, but the quantity and progressive motility of the spermatozoids were diminished. As expected, the transition from unsatisfactory spermatozoa values (during the non-breeding season) to satisfactory values occurred in the early to mid-fall months (September–October). This transition is characterized by a significant increase in the proportion of viable spermatozoa, increased concentration of spermatozoa and an increase in the progressive motility compared to the summer months. It is important to note that the BCS scores of the males on this study were somewhat constant between the summer months and the fall months, with the BCS ranging from 3.0–4.0. This body of evidence suggests that rams are affected by the seasonal effect of the photoperiod, but as opposed to females, they still have some reproductive capabilities, as long the body condition score of the animals remains acceptable (>2.5). Finally, to be considered satisfactory for use during the breeding season, a mature ram or ram lamb should have an ejaculate with normal appearance under microscopic examination; the ejaculate should have at least 50% spermatozoa with progressive motility and <20% abnormal spermatozoa. At the manual examination of the reproductive tract of the ram or ram lamb, the scrotal circumference should be >30 cm for ram lambs and >32 cm for mature rams (>2 years of age); animals must be free of any reproductive abnormality and be structurally sound, with good mobility [37] (see Table 1 for breed differences).

## 6. Ram Infertility

There are many causes associated with abnormal fertility in rams, some of which include poor libido, physical defects, disease, poor nutrition, injury, heat stress and age [48]. Proper ram management is crucial in the prevention and treatment of such causes of infertility, and it is necessary to implement practices to diagnose and treat potential issues in the early stages and, if treatment is not possible, to cull the animal.

Heat stress is an increasing problem for producers across the world. Heat stress can be induced with temperatures above 32 degrees Celsius for long periods of time or greater than 37 degrees Celsius for short periods of time. This results in an increased number of abnormal spermatozoa in the ejaculate, a phenomenon that can be observed 35 days post heat stress exposure [49,50,51]. Heat stress during the summer months can greatly affect the production of viable sperm and increase the percentage of unsatisfactory spermatozoa morphology, motility and concentration. The prevention of heat stress is critical when it comes to ram pre-breeding management. Some preventative practices include shearing the wool 4–6 weeks prior to the hot summer months (depending on the breed of the male) and providing adequate shade and clean, fresh, high-quality water. The negative effects of heat stress can potentially affect BSE evaluation results; therefore, it is recommended to perform the BSE four to eight weeks post exposure to extreme hot conditions [52,53].

Another important aspect that can affect fertility includes epididymitis. Epididymitis is a multifactorial clinical sign that can be observed by an increased size of the epididymis and adjacent structures. One of the diseases that commonly induces this condition is *Brucella ovis,* which is a common cause of infertility in rams and is more prominent in mature rams than in ram lambs. *Brucella ovis* is a reproductive disease that causes infertility in males, and occasionally abortion in females. The economic effects of *Brucella ovis* infection in males are due to subfertility and/or infertility in infected males, reducing conception rates in females [54,55]. According to data published by Van Metre et al. [3], from a subset sample of animals tested by ELISA for antibodies to *Brucella ovis*, 10% (233/2317) of animals had antibody titers indicative of infection. Additionally, the authors reported that 125 (53.6%) of these males had normal BSEs, with no apparent abnormalities in the testes and epididymis. Since this disease is an important reproductive disease and is considered zoonotic, BSEs should include testing for *Brucella ovis* pre-breeding, to identify the positive males either clinically or sub-clinically. Transmission of the disease occurs from ram to ewe or from ram to ram, causing infected rams to produce lower quality semen, resulting in sub-fertile or sterile rams, depending on the severity of the disease presentation. This disease can be initially diagnosed through scrotal palpation, assessing the epididymis and testicular tissue, and a blood test that must be submitted to a certified laboratory for diagnosis [52,53,54,55].

Another reproductive abnormality that can affect fertility is a disease commonly known as “pizzle-rot” or enzootic posthitis. Pizzle-rot is an infection of the sheath of the penis and is caused by the bacteria *Corynebacterium renale*. Pizzle-rot can also be caused by high protein diets that include a crude protein value higher than 16%. The presence of high protein diets results in an increased concentration of ammonia in the urine, causing the urine to become alkaline. This causes severe irritation and ulceration of the skin and mucosa around the preputial opening and penis. These ulcers and debris can also cause the formation and accumulation of crust and cellular debris around the prepuce, blocking the opening. This can result in pain and discomfort in the ram, resulting in an unwillingness to breed. In addition, rams with this disease should not be used for breeding until the ulcers have healed, as there have been reports of venereal transmission to ewes causing ulcerative vulvitis [21,40]. For a more extensive review of management of reproductive diseases in small ruminants, see Stewart and Shipley [56].

## 7. Ram and Ram Lamb Nutrition

Nutrition plays an important role in the reproductive processes, and it should be an important aspect to consider during the non-breeding season. From a practical standpoint, rams should be evaluated for body condition 6–8 weeks before the breeding season. In many cases, forage alone is not an adequate source of nutrition for growing ram lambs that are intended to be used for breeding. A recommended management practice for rams and ram lambs prior to the breeding season is to have a minimum body condition score of 3 (in a scale of 1–5, where 1 = emaciated and 5 = obese) [18,57]. In fact, Maurya et al. [57] demonstrated that rams with a pre-breeding BCS of 3 performed better in terms of reproductive efficiency as compared to animals with a BCS of 4 or animals with a low BCS, ≤2. Once rams are turned in with ewes, their feed intake decreases and they expend a lot of energy breeding the females. Rams and ram lambs show a dramatic body weight loss and consequently decrease in body condition score during the reproductive season, increasing the probability that the animal will be subsequently culled from the flock. Many rams will lose body weight and body condition score during a normal breeding season due to environmental factors and the quantity and quality of the pasture, which is often of low quality at that time of the year. In addition, the time spent on sexual behaviors to find a suitable ewe and mate reduces the amount of time available for feeding [58]. Many rams will lose between 10–15% of their body weight during a 45 day breeding period, and the loss can be greater if nutritional conditions are limited or they have large/difficult terrains to cover [59].

In terms of minerals, sheep can have limited access to a balanced mineral program or inadequate water consumption resulting in compromised ram growth and reproductive performance. Rams have a high requirement for zinc, selenium and cobalt for adequate semen quality [60,61,62]. The mineral profile in the western United States is varied across individual states, and many commercially available minerals are not adequately balanced to avoid deficiencies and maximize ram growth and breeding soundness. For example, Washington State is generally deficient in selenium, and the central region of the state is deficient in copper because of antagonist properties of molybdenum. However, most commercially available minerals for sheep only have 30–50 ppm selenium and no copper in the mineral profile [63].

Vitamins are another important class of nutrients, with important reproductive functions [64]. All sheep require vitamins A, D, and E in various quantities; it is recommended to consult a nutritionist to adequately formulate a balanced diet in line with the plant resources and needs of each particular breed. Specifically for male reproduction, vitamin E in addition to selenium deficiencies can cause degeneration of the testicular tissues, resulting in defective spermatogenesis due to their effect as an antioxidant and their role in prostaglandin synthesis and growth metabolism [64,65,66].

When rams are breeding, especially in multi-ram breeding pens, they will not leave the ewes in standing heat to travel great distances to have access to minerals or water. In addition, extra attention should be given to ram lambs in large breeding herds, not only because of the extra nutrition needed for growth, but also because their lack of experience often results in excess energy use in detecting heats or servicing ewes. Finally, it is important to note that rams and ram lambs have to be in optimal condition to reproductively perform with optimal results during the breeding season. The male contribution to the offspring is 50%, but in practical terms, there is a greater potential impact of the ram on the breeding season, because one male could affect a large group of females and greatly impact the reproductive, productive and economic efficiency and sustainability of the flock [66].

In sheep that are raised in extensive systems, either in rangelands or having access to grazing paddocks, parasitic diseases are an important cause of health problems for the animals. Depending on the region of the world and the climatic conditions, the species of parasites that could affect the gastrointestinal tract are different and vary according to the general management of the flock. In US systems, the most common parasites, associated with diarrhea, low ADG and feed efficiency, are *Trichostrongylus* spp. [67], *Nematodirus* spp., *Haemonchus* spp., *Strongyles* spp. [68], and coccidiosis caused by Eimeria spp. [69]. In terms of the pre-breeding management of the rams, it is recommended to use anthelmintic treatments 1–2 months before the breeding season. Selection of the anthelmintic drug should be in consultation with the veterinarian of the flock and or local agricultural authorities to avoid anthelmintic resistance. Additionally, other aspects to consider while managing parasitic diseases are specific diagnoses of the parasites that are present in the flock with fecal samples and treatment of the parasitic diseases using appropriate drugs, with adequate doses and frequency. The most sustainable approach for the flock is the rotation of the grazing paddocks (aiming to break the parasite cycle), strategic treatment of animals with anthelmintic drugs, and genetic selection of resistant animals [70,71,72]. 

Finally, in addition to the BSE and the assessment hoofs, thoracic and pelvic limbs integrity and overall health, one important aspect to consider managing ram and ram-lambs in range or extensive conditions is the appropriate mating ratios (number of females/male) and the socio-sexual interactions that occur between females and the male [72,73]. In general, it has been suggested that a mature ram (>2 years of age or with experience breeding ewes) can mate 30–50 ewes in range conditions, in contrast, for ram-lambs or animals around 1 year of age without experience breeding ewes, it is recommended 15–30 ewes per male [17,39]. It is important to note that animals managed in range and extensive conditions have increased activity detecting ewes in estrus and walking long distances. The food supply and source of nutrients can be highly variable in terms of the quality and quantity due to seasonal variations, and these animals are exposed to a great variety of climatic conditions and the presence of predators. All of these factors can impact the reproductive performance of the male and the pregnancy rate of the flock [74]. Therefore, it is recommended to frequently monitor the body condition score of the males during the breeding season, assess the hoof health and limb integrity and treat animals promptly when needed. 

## 8. Conclusions

The reproductive performance of a flock relies on many key factors to ensure success as well as to ensure economic profitability and sustainability. The reproductive performance of the flock relies on the fertility of the ewes and the rams equally. However, the selection criteria for the rams typically are based on phenotypic traits, and it is necessary to start selecting rams and ram lambs based on genetic/genomic information and reproductive traits. Implementation of successful reproductive programs in commercial sheep production farms should include adequate conditioning of the females and males during the off season to ensure optimal reproductive results at the end of the breeding season. The selection of animals for breeding should prioritize genotypic traits such as production variables (for instance, average daily gain, feed efficiency, market weight and carcass traits) as well reproductive variables (fertility, number of lambs weaned, etc.). Opportunities to improve the efficiency of the farm exist through the careful assessment and examination of the selection criteria of the males used on the farm. With recent advancements in genomic selection, it is possible to track and follow animals with desirable traits and cull animals that have genes detrimental to production.

## Data Availability

Not applicable.
